# Adipocyte PU.1 knockout promotes insulin sensitivity in HFD-fed obese mice

**DOI:** 10.1038/s41598-019-51196-8

**Published:** 2019-10-14

**Authors:** Denise E. Lackey, Felipe C. G. Reis, Roi Isaac, Rizaldy C. Zapata, Dalila El Ouarrat, Yun Sok Lee, Gautam Bandyopadhyay, Jachelle M. Ofrecio, Da Young Oh, Olivia Osborn

**Affiliations:** 1 Department of Medicine, University of California San Diego, 9500 Gilman Drive, La Jolla, CA 92093 USA; 2 Touchstone Diabetes Center, Department of Internal Medicine, University of Texas Southwestern Medical Center, 5323 Harry Hines Blvd, Dallas, Texas USA

**Keywords:** Type 2 diabetes, Type 2 diabetes

## Abstract

Insulin resistance is a key feature of obesity and type 2 diabetes. PU.1 is a master transcription factor predominantly expressed in macrophages but after HFD feeding PU.1 expression is also significantly increased in adipocytes. We generated adipocyte specific PU.1 knockout mice using adiponectin cre to investigate the role of PU.1 in adipocyte biology, insulin and glucose homeostasis. In HFD-fed obese mice systemic glucose tolerance and insulin sensitivity were improved in PU.1 AKO mice and clamp studies indicated improvements in both adipose and liver insulin sensitivity. At the level of adipose tissue, macrophage infiltration and inflammation was decreased and glucose uptake was increased in PU.1 AKO mice compared with controls. While PU.1 deletion in adipocytes did not affect the gene expression of PPARg itself, we observed increased expression of PPARg target genes in eWAT from HFD fed PU.1 AKO mice compared with controls. Furthermore, we observed decreased phosphorylation at serine 273 in PU.1 AKO mice compared with fl/fl controls, indicating that PPARg is more active when PU.1 expression is reduced in adipocytes. Therefore, in obesity the increased expression of PU.1 in adipocytes modifies the adipocyte PPARg cistrome resulting in impaired glucose tolerance and insulin sensitivity.

## Introduction

Insulin resistance is a characteristic defect in the majority of patients with type 2 diabetes mellitus (T2DM)^1^. Obesity is the major cause of insulin resistance in man and the increasing global obesity rates are tightly coupled to the parallel increase in global rates of T2DM^2^. Adipose tissue plays an important role in maintaining metabolic function and it is well described that chronic adipose tissue inflammation is an important cause of systemic insulin resistance.

PPARg is a master regulator of adipogenesis, and regulates the expression of many genes associated with metabolic pathways^3,4^. Indeed, PPARg expression in other cell types, such as fibroblasts and myoblasts, results in an adipocyte-like phenotype^5^. In contrast, the signal independent transcription factor PU.1, encoded by the gene *Pu1* or *Spi1*, is a key regulator of the hematopoietic lineage, especially of antigen presenting cells including macrophages, dendritic cells, and B cells. Binding of PU.1 to its recognition motifs facilitates chromatin opening and, together with DNA methyltransferases and other transcription factors, allows for signal-dependent transcription factor binding and transcription initiation^6,7^. PU.1 is also a potent re-programming factor where ectopic expression of PU.1 induces monocyte, macrophage, or dendritic cell morphology and function in fibroblasts and neural stem cells^8–11^.

More recently, it has been discovered that PU.1 is expressed by adipocytes, with increased expression in the obese state, particularly in the gonadal WAT depot^12,13^. PU.1 overexpression in 3T3-L1 preadipocytes inhibits the adipocyte differentiation process, partially through repression of C/EBPα and β transcriptional activity^12,13^. Conversely, PU.1 knockdown in differentiated 3T3-L1 or OP9-K adipocytes leads to increased insulin signaling with decreased pro-inflammatory cytokine expression and increased adipogenesis^13,14^.

Due to its role as a lineage-determining transcription factor, PU.1 has also been studied in the context of PPARg cistrome programming. For example, PU.1 overexpression caused a 75% loss of PPARg binding to target gene promoters with a global decrease in PPARg binding strength^15^. These studies suggest that increased adipocyte PU.1 expression in obesity could promote the proinflammatory, insulin resistant state. However, the role of PU.1 in adipocytes is poorly defined *in vivo* in the context of obesity. In the current studies, we generated adipocyte-specific PU.1 knockout (PU.1 AKO) mice to assess its role in adipogenesis, adipose tissue inflammation, and insulin resistance in the obese state. We show that PU.1 AKO mice fed high fat diet (HFD) have improved glucose tolerance and insulin sensitivity, with decreased adipose tissue inflammation, increased PPARg-target gene expression and decreased hepatic steatosis.

## Results

### Obesity results in increased PU.1 expression

**Figure 1:**
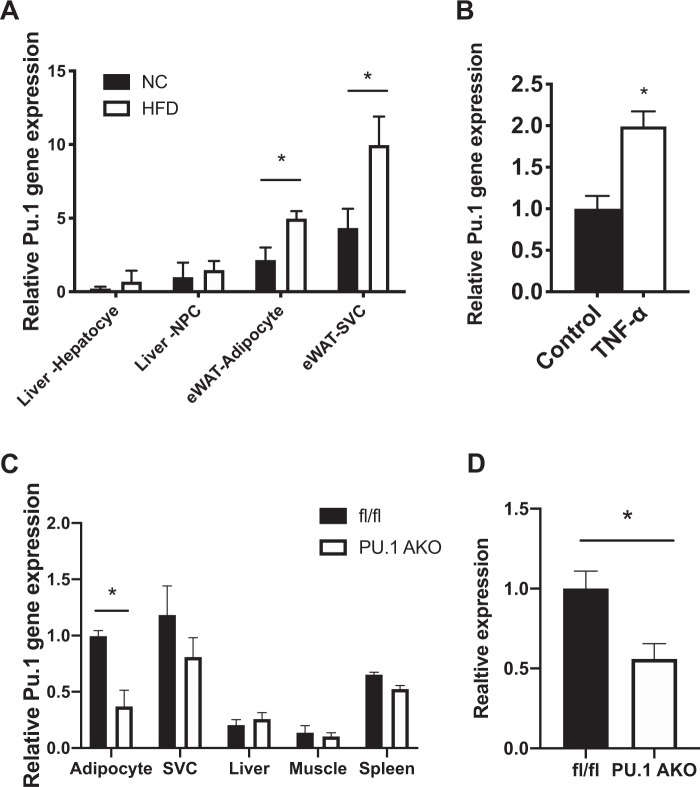
*Pu1* expression. (**A**) Relative *Pu1* expression in various cell types from normal chow (NC) and high fat diet (HFD)-fed mice. (**B**) Relative *Pu1* gene expression in 3T3-L1 adipocytes harvested 7 d post-differentiation, treated with or without TNFa for 48 h. (**C**) Relative *Pu1* expression in various cell types/tissues in fl/fl and PU.1 AKO mice after 14 wk HFD, normalized to adipocyte fl/fl expression. (**D**) Quantification of western blot detection of PU.1 in eWAT from fl/fl and PU.1 AKO mice, relative to HSP90 expression (see Supplemental Fig. 1C). Values are fold induction of gene expression normalized to the housekeeping gene *Rplp0* and expressed as mean ± SEM, n = 5 per group, *p < 0.05 comparing two groups using a t-test.

*PU.1* is highly expressed in adipose tissue with the stromal vascular cells (SVCs) largely contributing to the overall expression under lean conditions (Fig. 1A). However, in adipose tissue from HFD-fed obese mice the adipocyte levels of PU.1 are significantly induced in both SVCs and adipocytes (Fig. 1A). PU.1 is also expressed in the liver but expression levels are unchanged after HFD feeding in both hepatocytes and non-parenchymal cells (NPCs) (Fig. 1A). Treatment of differentiated 3T3-L1 adipocytes with TNF-alpha, a potent pro-inflammatory stimulus^16^, significantly increases PU.1 expression compared with control treated cells (Fig. 1B).

### PU.1 AKO tissue panel and genotyping

To investigate the role of adipocyte PU.1 in obesity-related insulin resistance, we generated adipocyte-specific PU.1 knockout (PU.1 AKO) mice by using adiponectin (*Adipoq*) Cre-mediated excision of PU.1 floxed alleles (*Pu1* fl/fl; *Adipoq-Cre*^+/−^). Floxed *Pu1* mice that do not express *Cre*-recombinase were used as controls, referred to as fl/fl (Fig. S1A,B). There was no significant difference in glucose tolerance between WT and adiponectin cre mice (Supplemental Fig. 2C). Under HFD conditions, PU.1 AKO mice show ~63% decreased *PU.1* mRNA expression in the eWAT adipocytes compared with fl/fl controls. *Pu1* expression is unchanged in SVC cells and other insulin responsive metabolic tissues (liver and skeletal muscle) and the immunologic tissue (spleen) in PU.1 AKO compared with WT mice (Fig. 1C). Western blotting of the eWAT adipose tissue confirmed significant reduction of PU.1 protein expression in PU.1 AKO mice compared with fl/fl controls (Fig. 1D and Supplemental Fig. 1C).

### PU.1 AKO results in improved glucose tolerance and insulin sensitivity

**Figure 2:**
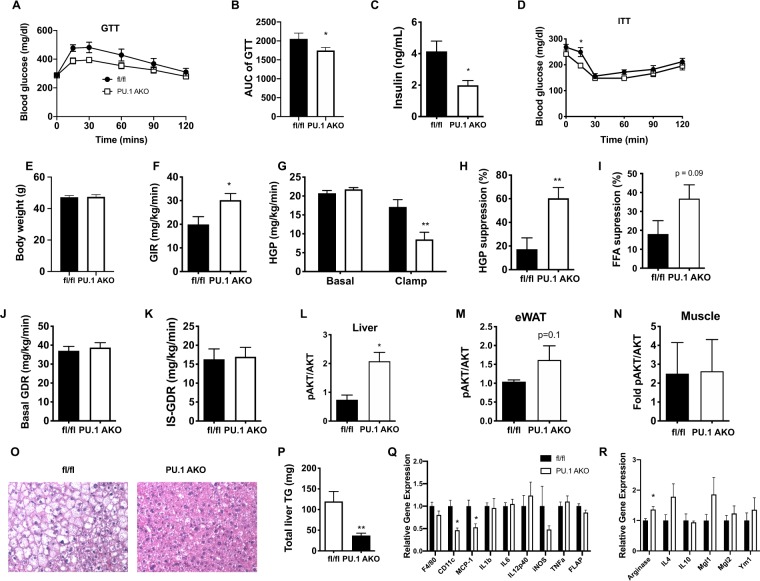
Improved glucose tolerance and insulin sensitivity in PU.1 AKO mice. (**A**) Glucose tolerance test (GTT) (**B**) Area under curve (AUC) of GTT. (**C**) Fasting insulin after 10 wks of HFD, and (**D**) insulin tolerance test after 11 wks of HFD in fl/fl and PU.1 AKO. (**E**) Body weight, (**F**) glucose infusion rate (GIR), (**G**) basal and clamp hepatic glucose production (HGP), (**H**) percent HGP suppression, (**I**) percent free fatty acid (FFA) suppression, (**J**) basal glucose disposal rate (GDR), and (**K**) insulin stimulated (IS)-GDR during hyperinsulinemic-euglycemic clamp after 12 wks HFD. Insulin-stimulated AKT phosphorylation in (**L**) liver, (**M**) eWAT, and (**N**) skeletal muscle 14 wk after HFD. (**O**) Representative H&E staining of paraffin embedded liver sections. (**P**) Triglyceride (TG) concentration in livers from HFD-fed fl/fl and PU.1 AKO mice. Expression of genes associated with pro-inflammation (**Q**) and anti-inflammation (**R**) in livers from HFD-fed fl/fl and PU.1 AKO mice. Values are expressed as mean ± SEM, n = 7–10 per group, *p < 0.05 by t-test.

To investigate the role of adipocyte PU.1 expression in the context of obesity and insulin resistance PU.1 AKO and fl/fl control mice were fed 60% HFD for up to 14 weeks, starting at 10 weeks of age. Obese, HFD fed PU.1 AKO mice showed improved glucose tolerance (Fig. 2A,B) with lower basal insulin levels compared to fl/fl control mice (Fig. 2C) and improved insulin sensitivity (Fig. 2D). Both fl/fl and PU.1 AKO mice become equally obese after consuming HFD (Fig. 2E). Lean PU.1 AKO mice did not show any differences in glucose or insulin tolerance compared to lean fl/fl mice, likely due to the observation that adipocytes from lean mice have very low levels of PU.1 (Fig. S2A,B). To quantify the tissue-specific responses to insulin, hyperinsulinemic-euglycemic clamp studies were performed in HFD fed PU.1 AKO and fl/fl controls. The amount of exogenous glucose required to maintain euglycemia (the glucose infusion rate (GIR)) was greater in PU.1 AKO mice, indicating improved insulin sensitivity in PU.1 AKO mice (Fig. 2F). The increased insulin sensitivity in PU.1 AKO mice was primarily due to an increased hepatic response to insulin, resulting in greater suppression of hepatic glucose production (HGP) during the clamp studies (Fig. 2G,H). There was also a trend (p = 0.09) towards greater suppression of free fatty acid (FFA) secretion from adipose tissue in PU.1 AKO mice (Fig. 2I). Basal and insulin-stimulated glucose disposal rates (GDR) were similar in WT and PU.1 AKO mice suggested there was no difference in muscle insulin sensitivity (Fig. 2J,K). We also conducted acute *in vivo* insulin stimulation studies and found a similar pattern as observed in the clamp studies with significantly increased pAKT activation in the liver, and a trend (p = 0.1) for improvement in eWAT in PU.1 AKO mice compared to fl/fl mice, with (Fig. 2L,M). There was no difference in pAKT levels in insulin-stimulated muscle from PU.1 AKO or WT mice (Fig. 2N). Histologic images of hemotoxylin and eosin stained liver sections indicate less fat accumulation in livers from HFD-fed PU.1 AKO mice with fewer ballooned hepatocytes (Fig. 2O) compared with fl/fl controls. In addition, liver triglyceride concentrations were also significantly decreased in PU.1 AKO mice (Fig. 2P). Gene expression of pro-inflammatory mediators was decreased in livers from obese PU.1 AKO mice compared to fl/fl mice, including the M1-like macrophage surface marker *Cd11c*, and chemokine Mcp1 **(**Fig. 2Q**)**. In addition, the M2-like macrophage-associated anti-inflammatory mediator *Arg1* was significantly increased in the livers of PU.1 AKO HFD mice compared to fl/fl controls (Fig. 2R).

### PU.1 AKO results in increased expression of adipogenic genes

**Figure 3:**
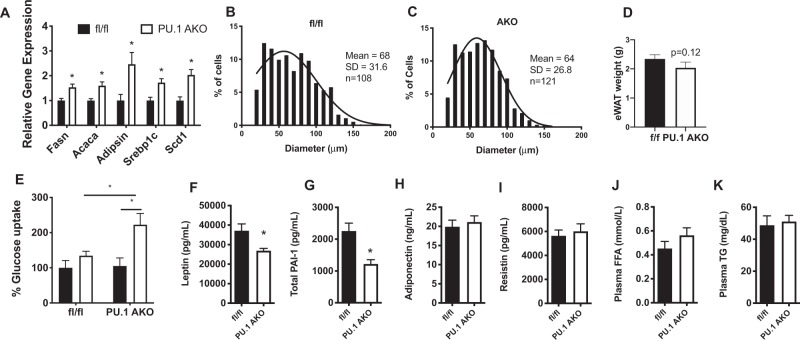
PU.1 negatively affects adipocyte metabolism. (**A**) Relative mRNA expression of adipogenic-associated genes in eWAT from fl/fl and PU.1 AKO mice after 14 wk HFD feeding and (**B**). Adipocyte diameter was quantified and sorted by bins with Lowess curve fitting in both f/f mice (**B**) and PU.I AKO mice (**C**). (**D**) Epididymal adipose weight. Glucose uptake, as a percent of basal ^3^H-2-deoxy-D-glucose uptake, in (**E**). Cultured primary adipocytes from eWAT from HFD-fed fl/fl and PU.1 AKO mice. Plasma concentrations of (**F**). leptin, (**G**). Total PAI-1, (**H**). Adiponectin, (**I**). Resistin, (**J**). Free fatty acids (FFA), and (**K**). Triglycerides (TG) in fl/fl and PU.1 AKO mice after 14 wk HFD feeding. Values are expressed as mean ± SEM, *p < 0.05 by t-test. All groups are significantly different from each other for each gene analyzed in B by 2-way ANOVA followed by Tukey’s post-hoc test. N = 10–14 per group in (**A**) and (**F**–**K**), n = 6–8 per group in (**B**–**E**).

In previous *in vitro* based experiments PU.1 has been shown to inhibit adipogenesis^12,17,18^. In agreement with these studies we also observed significantly greater levels of the adipogenic genes *Fasn*, *Acaca*, *Adipsin*, *Srebp1c* and *Scd1* in adipose tissue from PU.1 KO mice compared with fl/fl controls (Fig. 3A). Comparison of eWAT adipocyte size from HFD fed fl/fl and PU.1 AKO mice showed no significant difference in cell size with average cell diameter of 64 ± 26.8 μm in PU.1 KO mice vs 68 ± 31.6 μm vs. in fl/fl mice (Fig. 3B,C). Epididymal adipose tissue mass was slightly decreased in PU.1 KO mice (Fig. 3D) but this was not statistically significant (p = 0.12). To determine how PU.1 functionally affects adipocyte insulin sensitivity, we measured glucose transport in primary adipocytes. Cells from PU.1 AKO HFD mice are more insulin sensitive than adipocytes from fl/fl HFD mice, as demonstrated by greater insulin-stimulated glucose uptake (Fig. 3E). We also observed decreased concentrations of the circulating adipokines leptin and PAI-1 in PU.1 AKO mice while levels of adiponectin, resistin, FFA, and triglycerides were the same between the two groups (Fig. 3F–K). Together these data indicate that increased adipocyte expression of PU.1 in obese mice plays a role in the development of obesity-induced insulin resistance.

### PU.1 KO results in decreased adipose tissue inflammation

**Figure 4:**
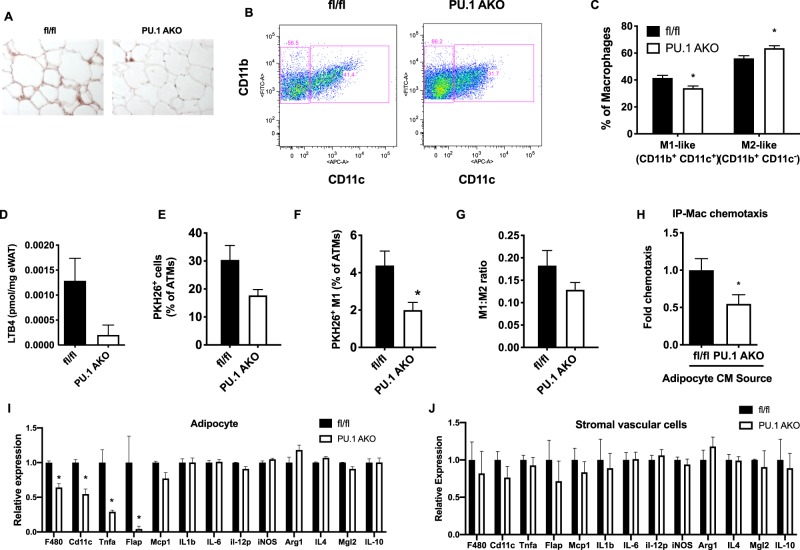
PU.1 affects eWAT inflammation in obesity. (**A**) Paraffin embedded eWAT sections were stained with the macrophage marker F4/80 by immunohistochemistry. (**B**) Flow cytometry gating strategy for analysis of F4/80^+^ ‘M1-like’ (F4/80^+^ CD11b^+^ CD11c^+^) and ‘M2-like (F4/80^+^ CD11b^+^ CD11c^+^) stromal vascular fraction from fl/fl and PU.1 AKO eWAT after 14 wk HFD feeding and (**C**). “M1-like” and “M2-like” macrophages. (**D**) LTB4 local concentration in eWAT in fl/fl and PU.1 AKO HFD fed mice. (**E**) PKH26 fluorescently-labeled total macrophage, (**F**). M1-like macrophage, and (**G**). Ratio of ‘M1-like:M2-like’ macrophages tracking in eWAT from fl/fl and PU.1 AKO recipient mice after 14 wk HFD feeding. (**H**) Chemotaxis of WT intraperitoneal-macrophages (IP-Mac) toward conditioned medium from fl/fl or PU.1 AKO cultured primary eWAT adipocytes from HFD mice. Analysis of Inflammation-associated gene expression from (**I**). Adipocytes or (**J**). Stromal vascular cells from eWAT from HFD fed fl/fl or PU.1 AKO mice. Values are expressed as mean ± SEM, *p < 0.05 by t-test (**D**–**G**), or ANOVA followed by Tukey’s post-hoc test. N = 4–8 per group in (**A**–**C**), 10–14 per group in (**D**,**H**–**J**) and n = 6–8 per group in (**E**–**G**).

We next studied the contribution of PU.1 expression on the inflammatory state of the adipose tissue in HFD-fed obese PU.1 AKO and fl/fl mice. We found decreased macrophage infiltration in eWAT in PU.1 AKO mice, as displayed in F4/80-stained immunohistochemistry sections, compared with fl/fl HFD-fed mice (Fig. 4A). FACs analyses of the eWAT SVCs revealed lower levels of M1-like/proinflammatory cells (F4/80^+^ CD11b^+^ CD11c^+^) and increased levels of M2-like/less inflammatory macrophages (F4/80^+^ CD11b^+^ CD11c^−^) in PU.1 AKO mice compared to fl/fl mice (Fig. 4B,C). In light of the decreased number of M1-like macrophages observed in PU.1 AKO eWAT compared with control and the decreased local production of the chemokine leukotriene LTB4 (Fig. 4D) we examined *in vivo* macrophage tracking in these mice (Fig. 4E–G). When PKH26-labeled WT peripheral blood monocytes were injected into fl/fl and PU.1 AKO HFD mice we found decreased PKH26-labeled cells in eWAT of PU.1 AKO recipient mice compared with WT (Fig. 4E) as well as decreased PKH26^+^ M1-like polarized cells (F4/80^+^ CD11b^+^ CD11c^+^) (Fig. 4F,G). Consistent with these *in vivo* macrophage tracking results we also observed similar effects in an *ex vivo* chemotaxis assays where conditioned medium (CM) from cultured primary PU.1 AKO HFD adipocytes showed a decreased effect to stimulate chemotaxis of WT IP macrophages than CM from fl/fl HFD adipocytes (Fig. 4H). Analysis of gene expression in eWAT from PU.1 AKO HFD mice showed significantly decreased adipocyte expression of inflammation-associated genes, including *F4/80*, *Cd11c*, *Tnfa* and decreased *Flap* (5-lipoxygenase-activating protein), which is an enzyme involved in the biosynthesis of LTB4^19^ (Fig. 4I) and no significant changes in inflammatory gene expression in the SVCs (Fig. 4I).

### Adipocyte specific ablation of PU.1 affects PPARg

**Figure 5:**
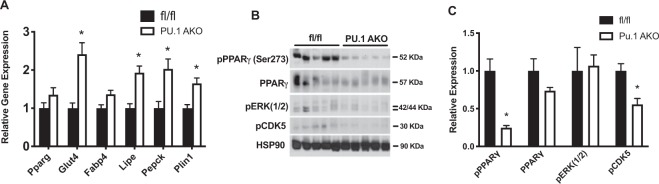
Adipocyte PU.1 expression negatively affects PPARg activity. PPARg target gene expression in (**A**). fl/fl or PU.1 AKO HFD eWAT. (**B**) Western blot of PPARg phosphorylation (Ser273),pERK and pCDK5 in eWAT from HFD-fed fl/fl and PU.1 AKO mice. (**C**) Quantification of western blot. Values are expressed as mean ± SEM, n = 5–10 per group, *p < 0.05 by t-test in (**A**,**C**).

Previous studies have shown that PU.1 globally dampens adipocyte PPARg binding^15^ so we went on to study how the absence of adipocyte PU.1 in obesity affects PPARg signaling. While the gene expression levels of transcription factor PPARg are unchanged we observed increased expression of PPARg target genes *Glut4*, *Lipe*, *Pepck*, and *Plin1* in eWAT from HFD fed PU.1 AKO compared with control fed mice (Fig. 5A). Phosphorylation at PPARg serine 273 is an additional mechanism by which PPARg target gene expression can be dysregulated^20,21^. While Ser-273 phosphorylation does not alter adipogenic capacity of PPARg^21^, target gene expression is dysregulated suggesting differential recruitment of co-regulators may drive the differences. In eWAT of PU.1 AKO HFD-fed mice we observed decreased pCDK5 associated with decreased phosphorylation of PPARg at serine 273 compared with fl/fl controls, (Fig. 5B,C and Supplemental Fig. S3) indicating that PPARg is more active when PU.1 expression is reduced in adipocytes.

## Discussion

PU.1 is also a potent re-programming factor which can confer macrophage-like phenotypic features and functions on other differentiated cell types through reprogramming and trans-differentiation^8–11^. Interestingly adipocyte expression of PU.1 increases significantly in the obese state^12^. In these studies, we generated adipocyte-specific PU.1 knockout mice and conducted detailed *in vivo* and *in vitro* studies of the role of adipocyte PU.1 in obesity-induced inflammation and insulin resistance. We found that adipocyte specific deletion of PU.1 results in systemic improvements in glucose tolerance and insulin sensitivity *in vivo*. Adipocyte specific KO of PU.1 resulted in decreased liver inflammation, decreased steatosis and significantly improved liver insulin sensitivity compared with fl/fl controls, as revealed by both hyperinsulinemic-euglycemic clamp and acute insulin signaling studies. It is well established that crosstalk between adipose tissue and liver plays a key role in the maintenance of metabolic homeostasis^22,23^. While PU.1 expression in the liver in unchanged in the PU.1 AKO mice, this suggests that signals from the adipose tissue drive changes in inflammation and insulin sensitivity in the liver. We propose the reduction in adipose tissue pro-inflammatory cytokines, chemokine, and leukotrienes, are likely to play a significant contribution in the resulting in decreased steatosis and improved insulin sensitivity observed in the liver of PU.1 AKO mice. Additionally, recent studies have highlighted the key role of adipose tissue macrophage derived exosomal miRNAs in the modulation of insulin sensitivity^24^ that could also be an important signal resulting in insulin sensitization in the liver in PU.1 KO mice.

Because of the improved metabolic phenotype in PU.1 AKO HFD mice, we sought to further examine the role of PU.1 in adipocytes during obesity. Previous *in vitro* studies have shown that PU.1 inhibits adipocyte differentiation^12,17,18,25,26^. In line with these studies we found PU.1 deletion in adipocytes resulted in increased expression of adipogenic genes (i.e. *Fasn*, *Acaca)*. While the *in vitro* studies show a clear role for PU.1 in adipogenesis, the reduction in adipocyte number and size from chronically fed HFD fed PU.1 AKO mice compared with floxed controls was very mild and not statistically significant. It is possible that other counter-regulatory genes are affected by PU.1 knockout *in vivo* or perhaps PU.1 expression in adipocytes does not increase immediately in response to HFD and the early phases of the PPARg-induced adipocyte differentiation course is not affected.

We conducted an *ex-vivo* glucose uptake assay in primary adipocytes and found HFD-fed PU.1 AKO adipocytes had significantly greater insulin-stimulated glucose uptake compared with HFD-fed fl/fl control mice. These results are consistent with previous *in vitro* studies showing increased insulin-stimulated glucose uptake in 3T3-L1 cells with PU.1 knockdown^13^.

As PU.1 is known as a master regulator of many types of immune cells, we reasoned that it likely plays a role in the adipose inflammation associated with obesity. In the obese state adipocytes produce and secrete proinflammatory signals that can regulate the recruitment and activation of adipose tissue macrophages^27–29^. When monocytes arrive in adipose tissue they differentiate into macrophages, which, in the inflammatory environment, polarize into pro-inflammatory “M1-like” macrophages, further producing more pro-inflammatory cytokines and chemokines. Adipocyte specific deletion of PU.1 resulted in reduced expression of pro-inflammatory genes in eWAT, decreased number of M1-like macrophages and decreased monocyte tracking to adipose compared with fl/fl control mice. *In vitro* chemotaxis assays confirmed decreased migration towards conditioned medium from primary PU.1 AKO adipocytes compared with controls. While levels of the classical chemotactic signal Mcp-1 were unchanged, levels of the leukotriene LTB4, a potent macrophage chemoattractant produced in adipose tissue during obesity^30^ were significantly lower in adipose tissue from PU.1 AKO mice.

Transcription factors PU.1 and PPARg both play key roles in regulating the expression of many genes associated with metabolic pathways and cell differentiation in adipose tissue. The PPARg cistrome between adipocytes and macrophages is predominantly cell type specific^31^. However when PU.1 is overexpressed at “macrophage levels” in mature adipocytes, PU.1 globally dampened adipocyte PPARg binding without affecting PPARg^15^. In agreement with these studies we found PU.1 deletion in adipocytes did not affect the expression of PPARg itself, but we observed increased expression of PPARg target genes *Glut4*, *Fabp4*, *Lipe*, *Pepck*, and *Plin1* in eWAT from HFD fed PU.1 AKO compared with control fed mice. Furthermore, in eWAT of PU.1 AKO HFD-fed mice we observed decreased phosphorylation at serine 273 compared with fl/fl controls, indicating that PPARg is more active when PU.1 expression is reduced in adipocytes. Therefore, in obesity the increased expression of PU.1 in adipocytes modifies the adipocyte PPARg cistrome. Siersbaek *et al*. have shown that the PPARg cistrome is different in adipocytes differentiated from various adipose depots including eWAT, scWAT, or BAT^32^. Interestingly in obesity PU.1 is predominantly increased in eWAT while largely unchanged in inguinal adipose tissue^13^ which suggests that the expression level of PU.1 in various adipose tissues depots may explain the differences in the PPARg cistrome and contribute to metabolic differences between the depots^33,34^.

In summary, adipocyte specific deletion of PU.1 in HFD-fed obese mice results in improved glucose tolerance and insulin sensitivity. In obesity, adipocyte levels of PU.1 are significantly increased and thus further understanding the aberrant transcriptional changes mediated by PU.1 may provide important therapeutic targets in the treatment of obesity-induced insulin resistance.

## Experimental Procedures

### Creation of adipocyte-specific PU.1 knockout mice

Mice with PU.1 floxed alleles were provided by Dr. David G. Tenen^35^. These mice were bred with transgenic mice containing Cre recombinase driven by the adiponectin promoter purchased from Jackson Laboratories (stock number 010803, Bar Harbor, ME) to create the following genotypes: PU.1 fl/fl (control), PU.1 fl/fl-AdipoqCre (PU.1 AKO). All experiments were approved by and conducted in accordance with the Animal Care Program at the University of California, San Diego.

### GTTs, ITTs, and hyperinsulinemic-euglycemic clamp study

Male fl/fl and PU.1 AKO mice were fed 60% high fat diet (HFD, D12492, Research Diets, New Brunswick, NJ). Glucose and insulin tolerance tests were performed on mice after 6 h fasting. Mice were injected IP with dextrose (1 g/kg, Hospira, Lake Forest, IL) for GTTs or with insulin (0.35 U/kg, Novolin R, Novo-Nordisk, Princeton, NJ) for ITTs. Blood was drawn at 0, 15, 30, 60, 90, and 120 min after injection for blood glucose determination using an Easy Step blood glucose monitor (Home Aid Diagnostics Inc, FL). Clamp studies were performed as previously described^36,37^ after 12 weeks of HFD feeding. Briefly, dual catheters (catalog no. MRE025, Braintree Scientific, Braintree, MA) were implanted in the right jugular vein, tunneled subcutaneously, and exteriorized at the back of the neck. Mice were allowed to recover for 4–5 days prior to the clamp procedure. After 6 h fasting, mice were placed in a Lucite restrainer (Braintree Scientific) and blood glucose was measured via tail nick. The experiment began with a constant infusion (5 μCi/hr) of D-[3-^3^H] glucose (PerkinElmer NEN Radiochemicals, Boston, MA) via the jugular vein cannulae. After 90 minutes of tracer equilibration and basal blood sampling at −10 and 0 min, insulin (8 mU/kg/min, Novo Nordisk) plus tracer (5 μCi/hr) and glucose (50% dextrose at variable rate, Hospira) infusions were initiated via the jugular vein cannulae simultaneously, with glucose infusion rate adjusted to reach a steady state blood glucose concentration (~120 min). Blood samples were collected via tail vein at −10, 0 (basal) 110, and 120 (steady-state) min to determine glucose-specific activity and insulin and free fatty acid (FFA) levels. Steady-state conditions (120 mg/dl ± 10 mg/dl) were confirmed at the end of the clamp by ensuring that glucose infusion rate and plasma glucose levels maintained constant for 30 min. Tracer-determined hepatic glucose production (HGP) and glucose disposal rate (GDR) were calculated at the basal state and during the steady-state portions of the clamp by using the Steele equation for steady-state conditions^38^. At steady state, the rate of glucose disappearance (total GDR) is equal to the sum of the rate of endogenous glucose production (i.e., HGP) plus the exogenous (cold) GIR. The IS-GDR is equal to the total GDR minus the basal glucose turnover rate.

### Gene expression

Epididymal adipose tissue (eWAT) was fractionated into adipocytes and the stromal vascular fraction (SVF) and liver fractionated into hepatocytes and non-parenchymal cells as described previously^37,39^. RNA extracted, converted to cDNA and relative expression analyzed by qPCR. Gene expression was calculated after normalization to the housekeeping gene *Rplp0* using the Δ^ΔCt^ method. Gene expression was calculated relative to fl/fl (tissue) or control (cell culture) samples. Primer sequences used to measure gene expression are listed in Table S1 in the supplemental material.

### SVC isolation and flow cytometry

Epididymal WAT was isolated from mice and minced in PBS containing 0.5% (w/v) FA-free BSA. Collagenase (C0130, Sigma) was added and tissue was incubated for 30 min at 37 °C while shaking. Cells were strained through a 100 μm cell strainer to remove tissue debris and separated by centrifugation into adipocytes and SVCs. The pelleted SVC fraction was analyzed by FACS. SVCs in single-cell suspension were incubated with purified CD16/CD32 (BD Biosciences, San Diego, CA) to block non-specific antibody binding and with Zombie Aqua Fixable Viability Dye (BioLegend, San Diego, CA) to discriminate live cells from dead cells. Cells were incubated with the following antibody-fluorochrome conjugates for extracellular marker recognition: CD11b-FITC (M170) and CD11c-APC (HL3) (from BD Biosciences, San Jose, CA), F4/80-PE-Cy7 (BM8, from eBiosciences, San Diego, CA).

### Primary adipocyte culture

Primary adipocytes isolated from eWAT were cultured as previously described^36^. Briefly, the adipocyte layer was washed in Krebs-Ringer solution (0.12M sodium chloride, 4.7 mM potassium chloride, 2.5 mM calcium chloride, 1.2 mM magnesium sulfate, 1.2 mM potassium phosphate, and 20 mM HEPES, pH 7.4, supplemented with 1% FA-free BSA, 200 nM adenosine, and 2 mM glucose) then resuspended in DMEM medium containing 4.5 mM glucose and supplemented with 4% FA-free BSA, 100 nM adenosine, 10 mM HEPES, Penicillin, and streptomycin. The cells were cultured at 37 °C, 5% CO_2_ and conditioned medium was collected after 24 h.

### Glucose uptake assays in primary adipocytes

Glucose uptake was measured after 24 h serum starvation in primary adipocytes^40^. Briefly, cells were equilibrated in Hepes-Salt buffer (10 mM Hepes, 40 mM potassium chloride, 125 mM sodium chloride, 0.85 mM potassium phosphate monobasic, 1.25 mM sodium phosphate dibasic, 1 mM magnesium chloride, 1 mM calcium chloride, 0.1% fatty acid-free BSA) for 2 h for glucose starvation, treated with or without 100 nM insulin for 20 minutes followed by 2 μCi ^3^H-2-deoxy-D-glucose for 10 minutes at 37 °C. The glucose uptake was halted by washing with ice cold PBS. The cells were lysed with sodium hydroxide, protein concentration measured, samples were neutralized with hydrochloric acid, and samples were counted in scintillation fluid.

### Histology and immunohistochemistry

Paraffin-embedded WAT and liver tissue sections were stained with hematoxylin and eosin (H&E). Anti-F4/80 antibody (Abcam, Cambridge, MA) was used for immunohistochemistry staining by the UCSD Histology and Immunohistochemistry Core. For eWAT adipocyte sizing, at least 100 cells from 3–5 images for each section were measured using CellProfiler software (v 2.1.1), using the Adipocyte H&E Cell Profiler Pipeline. The cell diameter was calculated for each cell and measured 100–300 cells per slide (3–5 fields). Pixel area per cell was converted to pixel diameter and subsequently converted to μm. Cells were then binned by diameter into 10 μm groups^41^.

### *In vivo* monocyte tracking assay

*In vivo* monocyte tracking was performed as previously described^42^. Briefly, leukocytes pooled from 10-wk-old male C57BL/6 mice bled from the retro-orbital sinus were subjected to red blood cell lysis. Isolated monocytes were washed in RPMI-1640 medium, counted, and suspended in 2 ml diluent solution C included in the PKH26 labeling kit (Sigma, St. Louis, MO) per 5 × 10^6^ to 10 × 10^6^ cells. 2 ml PKH26 diluted to 2 × 10^−3^ mol/L in diluent solution C was added to the monocytes and mixed. Monocytes were incubated in the PKH26-containing solution for 10 min in the dark at room temperature. Staining was halted by the addition of medium containing 10% FBS and monocytes washed in PBS and resuspended in serum containing medium. PKH26-labeled monocytes were counted and 1 × 10^6^ cells were suspended in 0.2 ml PBS and injected retro-orbitally into each recipient mouse (HFD-fed fl/fl or PU.1 AKO mice). SVCs were isolated from recipient mice 5 days post injection, eWAT, stained, and analyzed by FACS.

### *In vitro* IP-macrophage chemotaxis assay

Thioglycollate-elicited IP-macrophages were isolated from 8–10-wk-old male C57BL/6 mice and cultured for 3 days, as previously described^43^. For migration assays, macrophages were place in the upper chamber of an 8 μm polycarbonate transwell filter (24 well plate), while medium containing conditioned medium, diluted 1:200, from PU.1 AKO or fl/fl primary HFD adipocytes cultured for 24 h was placed in the lower chamber. After 3 h migration, cells were formalin-fixed and stained with 4′, 6-diamidino-2-phenylindole and observed. Successful macrophage migration was determined by macrophage migrating to the opposite side of the filter.

### 3T3-L1 *in vitro* studies

Cells were plated in 24 well plates with 1 × 10^5^ cells per well and treated for 48 h with 10 ng/ml TNF-a (Cell Signaling Technology, Danvers, MA) 1 d post-transfection.

### Western blots

Tissues were lysed in RIPA buffer, and concentration of protein lysates were measured using BioRad Protein Assay. Equal concentrations of protein were loaded and electrophoresed on 4–15% TGX Criterion gels (BioRad), transferred to PVDF membranes, and immunoblotted. Antibodies used were PU.1 (2266S, rabbit polyclonal, Cell Signaling), pSer 473 AKT (rabbit monoclonal, Cell Signaling), panAKT (mouse monoclonal, Cell Signaling), pSer 273 PPARg (bs-4888R, rabbit polyclonal, Bioss), PPARg (sc-7196, rabbit polyclonal, Santa Cruz), HSP 90α/β (sc-7947, rabbit polyclonal, Santa Cruz), Phospho-p44/42 MAPK (ERK1/2)(Thr202/Tyr204)(9101, Cell signaling), p-Cdk5(ser159), sc-12919, Santa Cruz), followed by appropriate HRP-linked secondary antibody. SuperSignal West Pico Chemiluminescent substrate (Thermo Scientific) was used to develop blots. Band intensity was measured using NIH ImageJ software.

### Determining adipocyte cell size

Adipose tissue was dissected, fixed, and embedded in paraffin, sectioned and stained with H&E. Images were captured using a NanoZoomer slide scanner system with NanoZoomer Digital Pathology software (Hamamatsu). Images were analyzed and cell diameter measured using ImageJ software and presented as number of cells in each cell size category.

### Plasma measurements

Plasma leptin, resistin, and PAI-1 were measured using a Milliplex Multiplex assay (Millipore, Billerica, MA). Plasma adiponectin was measured using ELISA (Millipore), plasma insulin was measured by ELISA (ALPCO). Plasma triglycerides and free fatty acids were measured enzymatically (NEFA C, Wako Chemicals).

### LTB4 measurement

LTB4 was measured in eWAT from fl/fl and PU.1 AKO mice after 14 wk HFD as previously described^44^.

### Statistical analysis

For two-group comparisons, data were analyzed by Student’s t-test. For two-way comparisons over time (GTT and ITT), data were analyzed by 2-way ANOVA for repeated measures with Sidak post-hoc test. For comparisons of more than two groups, data were analyzed by two-way ANOVA followed by Tukey’s post-hoc test. For multiple two-group comparisons, data were analyzed by multiple t-test followed by the Holm-Sidak method to correct for multiple comparisons. *P* < 0.05 was considered statistically significant. Using GraphPad Prism 6.0 software (San Diego, CA).

## Supplementary information

Supplemental Materials
