# Supplementary material for: Adipocyte PU.1 knockout promotes insulin sensitivity in HFD-fed obese mice
Source: Sci Rep. 2019 Oct 14;9:14779. doi: 10.1038/s41598-019-51196-8 (PMC6791934; doi:10.1038/s41598-019-51196-8)
Supplement: Supplementary file 1 — Supplemental Materials [file 41598_2019_51196_MOESM1_ESM.pdf]

Supplemental Figures

Title: Adipocyte PU.1 knockout promotes insulin sensitivity in HFD-fed obese mice

Denise E. lackey[1], Felipe C. G. Reis[1] Roi Isaac[1], Rizaldy C Zapata[1], Dalila El Ouarrat[1], Yun Sok Lee[1], Gautam Bandyopadhyay[1], Jachelle Ofrecio[1], Da Young Oh[2], Olivia Osborn[1]

1. Department of Medicine, University of California San Diego, 9500 Gilman Drive, La Jolla, CA, 92093, USA.
2. Department of Internal Medicine, University of Texas Southwestern, 5323 Harry Hines Blvd, Dallas, Texas.

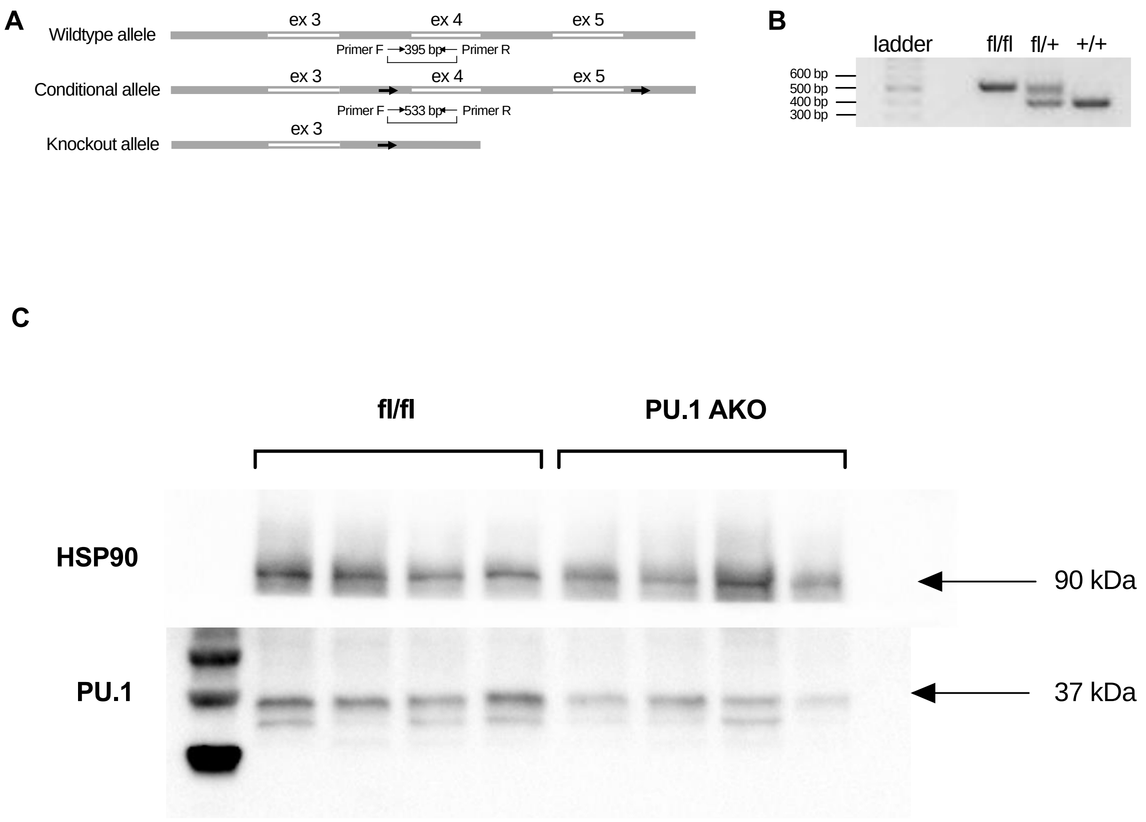

Figure S1. PU.1 targeting strategy in mice. A. Wild type, floxed, and deleted *Pu1*gene loci. Primers used to distinguish WT from PU.1 floxed alleles and sizes of the expected PCR products are indicated. B. Genotyping results for wild type +/+, fl/+, and fl/fl mice from tail biopsies. C. Western blot analysis of PU.1 expression in adipose tissue from fl/fl and PU.1 adipose specific KO (AKO) mice.

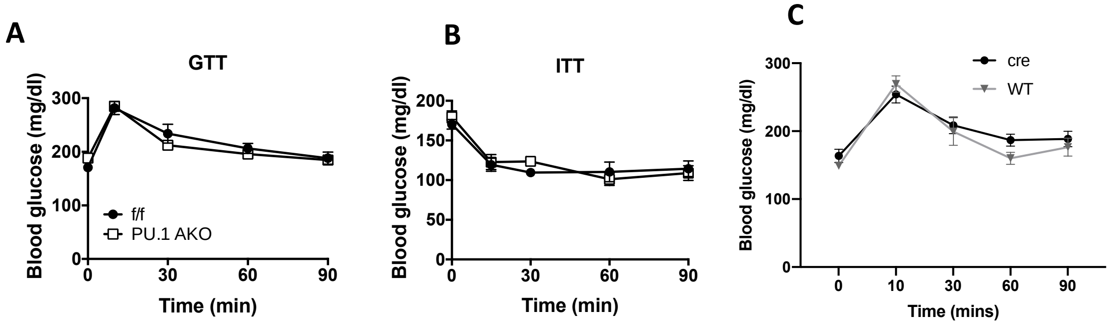

*

Figure S2. A) glucose tolerance and B) insulin sensitivity in lean, normal chow fed PU.1 AKO compared with controls, age 20 wks. Values are presented as mean ± SEM, n = 10-14 for A, n = 6 or 11 for B. C. Glucose tolerance test on Adiponectin cre and WT control, 12 week old male mice fed normal chow. There was no significant difference in glucose tolerance between the adiponectin-cre mice and WT controls, n=6 per group.

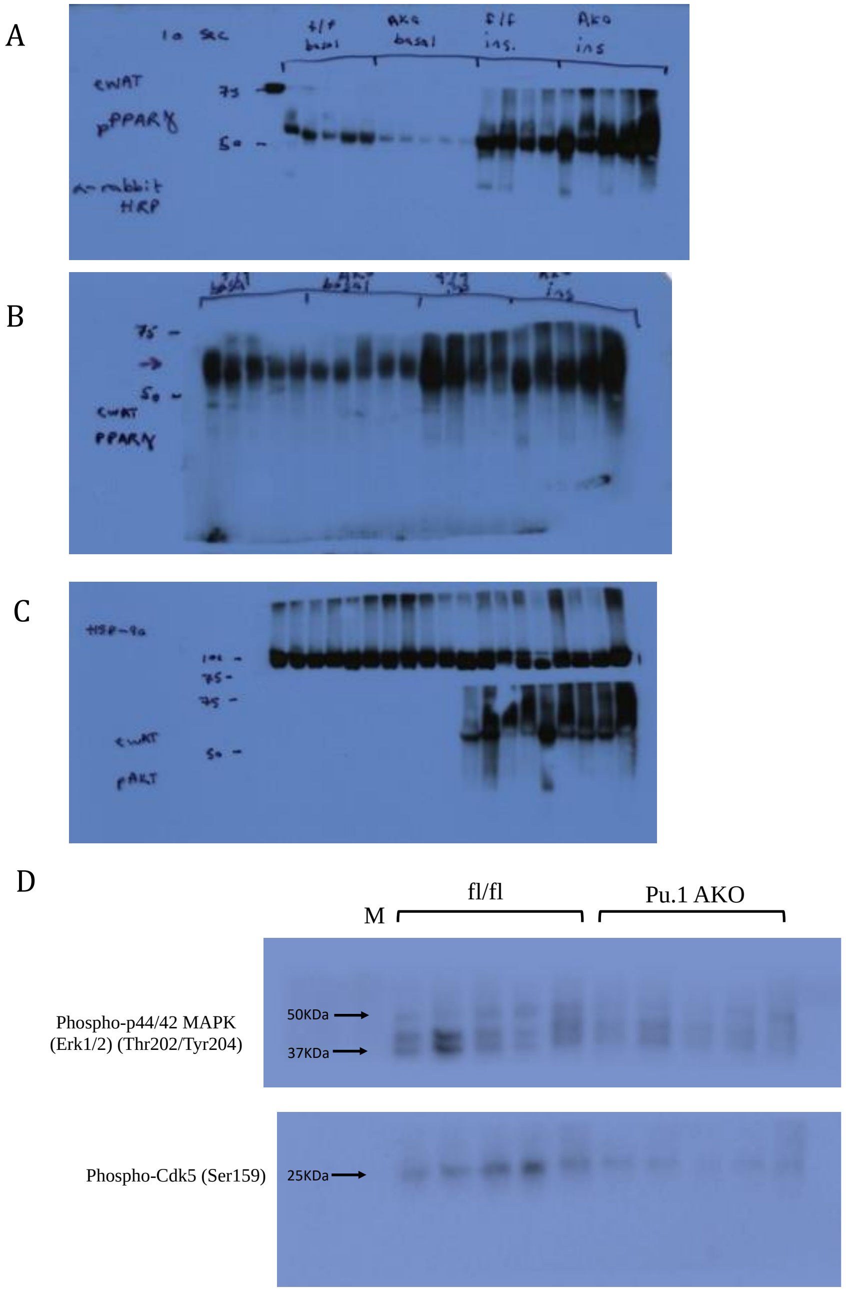

Figure S3. Full-length Western blots of A.PPARg phosphorylation (Ser273), B. total PPARg and C. HSP90, D. ERK1/2 (Thr202/Tyr204) and Phospho-Cdk5 (ser159) in epididymal adipose tissue from HFD-fed fl/fl and PU.1 AKO mice.

Table S1. Sequences of primers used for gene expression qPCR in this study.

Gene name	Forward primer (5'→3')	Reverse primer (5'→3')
36b4	TGGCCAATAAGGTGCCAGCTGCTG	CTTGTCTCCAGTCTTTATCAGCTGCAC
Acaca	CTGACGTATACTGAACTGGTGTTGGATG	TTTCCAGGCTACCATGCCAATCTC
Adipsin	CATGCTCGGCCCTACATGG	CACAGAGTCGTCATCCGTCAC
Arginase	ATGGAAGAGACCTTCAGCTAC	GCTGTCTTCCCAAGAGTTGGG
Cd11c	ACACAGTGTGCTCCAGTATGA	GCCCAGGGATATGTTCACAGC
F4/80	CTTTGGCTATGGGCTTCCAGTC	GCAAGGAGGACAGAGTTTATCGTG
Fabp4	GGATTTGGTCACCATCCGGT	CCAGCTTGTCACCATCTCGT
Fasn	CCAGACAGAGAAGAGCCATGGAGG	CCAATGAGGTTGGCCCAGAACTCC
Flap	GCATGAAAGCAAGGCGCATAA	GGTACGCATCTACGCAGTTCT
Glut4	CAATGGTTGGGAAGGAAAAGGGCTA	GTAGGCGCCAATGAGGAACCGTC
Il1b	AAATACCTGTGGCCTTGGGC	CTTGGGATCCACACTCTCCAG
Il4	ATGGAGCTGCAGAGACTCTT	AAAGCATGGTGGCTCAGTAC
Il6	CCAGAGATACAAAGAAATGATGG	ACTCCAGAAGACCAGAGGAAAT
Il10	TGAATTCCCTGGGTGAGAAG	TCACTCTTCACCTGCTCCACT
Il12p40	CCAGAGACATGGAGTCATAG	AGATGTGAGTGGCTCAGAGT
Lipe	GGGAGCTCCAGTCGGAAGA	AACAGTTGGCCTAGGGTTGG
Mcp1	AGGTCCCTGTCATGCTTCTG	GCTGCTGGTGATCCTCTTGT
Mgl2	CAGAACTTGGAGCGGGAAGAG	TTCTTGTCACCATTTCTCATCTCCT
iNOS/Nos2	GAGGCCCAGGAGGAGAGAGATCCG	TCCATGCAGACAACCTTGGTGTTG
Pck1	ATGAAGTTTGATGCCCAAGGCAAC	GGATTTGTCTTCACTGAGGTGCC
Pgc1a	TATGGAGTGACATAGAGTGTGCT	CTGGGCAAAGAGGCTGGTC
Plin1	TCCACCCAGTTCACAGCTGC	GATGCTGTTCCTGGCGCTTC
Pparg	GCATGGTGCCTTCGCTGA	TGGCATCTCTGTGTCAACCATG
Pu1	CCTCGATACTCCCATGGTGC	CCAGCAGGAACTGGTACAGG
Scd1	TTCTTGCGATACACTCTGGTGC	CGGGATTGAATGTTCTTGTCGT
Srebp1c	GCCGTGGTGAGAAGCGCACAGCCC	CAAGACAGCAGATTTATTCAGCTTTGC
Tnfa	CCAGACCCTCACACTCAGATC	CACTTGGTGGTTTGCTACGAC
Ym1	GGGCATACCTTTATCCTGAG	CCACTGAAGTCATCCATGTC
